# Association of baPWV and CD34^+^ progenitor-derived exosomal hsa_circ_0093884 with Alzheimer’s disease: mechanistic insights into the miR-375/SIX4 axis

**DOI:** 10.3389/fcell.2026.1764767

**Published:** 2026-05-14

**Authors:** Ying Chen, Yangyang Huang, Bei Gao, Yuhao Zhao, Lu Wei, Guoxin Ye, Shuyan Chen, Xibao Shi, Ruiliang Wang, Fei Wang

**Affiliations:** 1 Department of Geriatrics, Xinhua Hospital, Shanghai Jiaotong University School of Medicine, Shanghai, China; 2 Department of Gastroenterology, Shanghai General Hospital, Shanghai Jiao Tong University School of Medicine, Shanghai, China; 3 Department of Cardiology, Ruijin Hospital Affiliated to Shanghai Jiaotong University School of Medicine, Shanghai, China

**Keywords:** Alzheimer’s disease, circRNA, endothelial progenitor cell–derived exosomes, geriatric assessment, pulse wave velocity, vascular aging

## Abstract

**Background:**

Vascular aging plays a key role in the pathogenesis of Alzheimer’s disease (AD). This study evaluated the diagnostic value of arterial stiffness and endothelial progenitor CD34^+^ progenitor-derived exosomal circRNAs (EPC-Exos circRNAs) in AD, and examined their associations with Comprehensive Geriatric Assessment (CGA) measures.

**Methods:**

We measured brachial-ankle pulse wave velocity (baPWV) and conducted CGA in subjects with AD (n = 58), mild cognitive impairment (MCI, n = 31), and non-cognitive impairment (NCI, n = 36). Plasma levels of SIRT1-derived circRNAs (including hsa_circ_0093884) within EPC-Exos were quantified. Logistic regression and ROC curve analyses were used to assess diagnostic performance, with internal validation performed using bootstrapping (1,000 resamplings) to evaluate model stability. Aβ1-42-treated SH-SY5Y cells were used as a neuronal model to validate the downstream neuroprotective effects of circRNA. The miR-375/SIX4 regulatory axis was investigated using bioinformatics prediction, biotin-RNA pull-down, dual-luciferase reporter assays, and co-transfection experiments.

**Results:**

Compared to MCI and NCI groups, AD patients exhibited significantly higher baPWV and lower levels of EPC-Exosomal hsa_circ_0093884 (*P* < 0.01). A composite model incorporating baPWV, hsa_circ_0093884, and CGA scores showed excellent diagnostic performance (AUC = 0.943; 95% CI: 0.881–1.000), with the robustness of key predictors confirmed by bootstrap validation. In Aβ1-42-treated SH-SY5Y cells, overexpression of hsa_circ_0093884 increased cell viability and reduced apoptosis, whereas knockdown further decreased viability and promoted apoptosis. Mechanistically, hsa_circ_0093884 directly interacted with miR-375 and regulated SIX4 expression.

**Conclusion:**

EPC-Exosomal hsa_circ_0093884 and baPWV are valuable diagnostic biomarkers associated with Alzheimer’s disease. The hsa_circ_0093884/miR-375/SIX4 axis offers a novel therapeutic target, supporting a potential vascular-neuronal axis connecting vascular aging to neurodegeneration.

## 1 Introduction

Vascular aging is increasingly recognized as a key contributor to the pathogenesis of Alzheimer’s disease (AD), characterized by arterial stiffening, endothelial dysfunction, and impaired vascular reactivity. Clinical studies have consistently demonstrated that pulse wave velocity (PWV), the gold-standard marker of arterial stiffness, is significantly elevated in patients with AD and mild cognitive impairment (MCI) compared with cognitively normal individuals (Rivera-Rivera et al., 2021). Specifically, brachial-ankle PWV (baPWV), a widely used clinical modality, has shown strong correlations with cognitive decline (Fukuhara et al., 2006; Hughes et al., 2014; Jochemsen et al., 2015). These findings suggest that vascular aging, as reflected by baPWV, may serve as a valuable biomarker associated with cerebrovascular injury in neurodegenerative disorders.

Concurrently, endothelial dysfunction aggravates blood–brain barrier (BBB) disruption and neuroinflammation, a process characterized by the diminished reparative capacity of CD34^+^ progenitor cells (often referred to as endothelial progenitor cells, EPCs) (Zhang et al., 2018; Christov et al., 2008; Tarawneh et al., 2022). Studies indicate that circulating CD34^+^ progenitors are inversely associated with AD risk and negatively correlated with established AD biomarkers, such as Aβ1-42, as well as with aging (Nation et al., 2018; Maler et al., 2006; Custodia et al., 2021). Emerging evidence extends this vascular paradigm to EPC-derived exosomes (EPC-Exos). These nanoscale vesicles are capable of traversing the BBB and act as messengers in a potential “vascular-neuronal axis,” delivering bioactive cargos from the peripheral circulation to the central nervous system (Akhter, 2018). Among these cargos are circular RNAs (circRNAs), which possess exceptional stability and modulate gene expression via competitive endogenous RNA (ceRNA) networks. In our previous work, we identified five SIRT1-derived circRNAs, notably hsa_circ_0093884, that are abundantly packaged in EPC-Exos (Zhao et al., 2024), suggesting their potential involvement in AD-related pathology via this cross-talk mechanism.

Given the multifactorial nature of AD, comprehensive evaluation frameworks are essential. The Comprehensive Geriatric Assessment (CGA), which integrates somatic, functional, and psychosocial domains, provides a holistic measure of aging-related vulnerability (Schippinger, 2022). Importantly, up to 35%–40% of dementia cases may be attributable to modifiable risk factors amenable to lifestyle-based interventions (Livingston et al., 2020). These insights underscore the value of combining vascular biomarkers—such as baPWV and EPC-Exos–derived circRNAs—with CGA-derived metrics to improve diagnostic accuracy and risk stratification for AD.

In this study, we aimed to evaluate the diagnostic value of baPWV, EPC-Exos–derived circRNAs, and CGA as minimally invasive biomarkers associated with AD. We hypothesized that these markers not only reflect vascular aging but also provide mechanistic insights into AD pathogenesis—specifically regarding the neuroprotective role of vascular-derived circRNAs—thereby facilitating clinical diagnosis and improving risk assessment.

## 2 Materials and methods

### 2.1 Study participants and clinical data

This single-center, cross-sectional study enrolled geriatric inpatients at Xinhua Hospital, Shanghai Jiao Tong University School of Medicine, between June 2021 and June 2023. Participants underwent comprehensive clinical and neuropsychological assessments conducted by two experienced geriatric physicians with neurology expertise. Patients were selected based on predefined inclusion and exclusion criteria. Exclusion criteria were: (1) large-vessel stroke (small-vessel ischemic changes permitted); (2) major neurological diseases other than Alzheimer’s disease (AD) that could affect cognition; (3) organ failure, active cancer, uncontrolled psychiatric disorders, or a history of drug abuse or heavy alcohol consumption within the past 10 years. AD diagnosis followed the 2011 National Institute on Aging–Alzheimer’s Association (NIA-AA) criteria for probable AD dementia. Mild cognitive impairment (MCI) was defined according to Petersen (2004), and the non-cognitively impaired (NCI) control group comprised individuals deemed cognitively normal based on objective neuropsychological assessments. This study was approved by the Ethics Committee of Xinhua Hospital Affiliated to Shanghai Jiao Tong University School of Medicine (Approval No.: XHEC-WJW-2020-037). Each participant signed a written informed consent form.

Neuropsychological evaluations covered executive function, attention, language, visuospatial ability, and verbal and visual memory, using standardized tools including the Mini-Mental State Examination (MMSE), Montreal Cognitive Assessment (MoCA), and Clinical Dementia Rating (CDR). Comprehensive Geriatric Assessment (CGA) captured four domains: physical health, functional status, mental health, and socio-environmental factors. Instruments included the Frail Scale, Activities of Daily Living (ADL), Short Physical Performance Battery (SPPB), Pittsburgh Sleep Quality Index (PSQI), Nutritional Risk Screening 2002 (NRS 2002), Patient Health Questionnaire-9 (PHQ-9), and Generalized Anxiety Disorder scale (GAD-7). Vascular risk factors, including hypertension, coronary heart disease, and diabetes, were documented and categorized as single or multiple conditions.

### 2.2 Vascular assessment

Brachial–ankle pulse wave velocity (baPWV) was assessed using the VP-1000 (BP203RPE-III) device (Omron Colin, Japan). Participants rested in the supine position for at least 5 min before blood pressure cuffs were applied to both upper arms and ankles. The mean value of bilateral baPWV measurements was used for analysis. All assessments were performed by a trained operator blinded to clinical information. Carotid intima–media thickness (IMT) was evaluated using a Logiq E9 color Doppler ultrasound system equipped with an 8-12 MHz linear probe. IMT was measured at the distal common carotid artery, approximately 1 cm proximal to the carotid bifurcation.

### 2.3 Isolation of EPC-Derived exosomes

Peripheral venous blood (2-3 mL) was collected from all participants after an overnight (8 h) fast. Samples were processed within 2 h. Plasma was first centrifuged at 3,000 × g for 10 min at 4 °C, followed by a second centrifugation at 10,000 × g for 20 min to remove residual cells and debris. The supernatant was diluted with PBS and subjected to exosome isolation using a YeaSen plasma exosome extraction kit according to the manufacturer’s protocol. Exosome concentration was quantified using the BCA assay. For the enrichment of CD34^+^ progenitor cell-derived exosomes (predominantly EPCs), 1,000 μg of total plasma exosomes were incubated with 5 μg of biotinylated anti-CD34 antibody and rotated overnight at 4 °C. Streptavidin-coated magnetic beads were then added, followed by a 1-h incubation at room temperature. After magnetic separation and washing, CD34^+^ exosomes were collected. The pH of the final exosome preparation was adjusted to neutral using 10% Tris (pH 9.0).

### 2.4 Transmission electron microscopy (TEM)

Isolated exosomes (20 μL) were placed onto 200-mesh copper grids (Ted Pella, 01800) and allowed to adsorb for 5 min at room temperature. Excess liquid was removed using filter paper, followed by negative staining with 2% uranyl acetate (Sigma-Aldrich, 73943) for 1 min. Grids were air-dried for 20 min before imaging with a Hitachi HT-7700 transmission electron microscope operated at 100 kV.

### 2.5 Nanoparticle tracking analysis (NTA)

Ten microliters of isolated exosomes were diluted in 1× PBS and analyzed using a ZetaView PMX120-Z instrument (Particle Metrix, Germany) to determine particle size distribution and concentration. The system was calibrated using 110 nm polystyrene beads, and all measurements were performed at a controlled temperature of 23–30 °C.

### 2.6 Western blot analysis

Exosomal proteins were extracted using RIPA lysis buffer, and protein concentration was determined using a BCA Protein Assay Kit. To ensure proper normalization, equal amounts of total protein (20 μg) were loaded into each lane and separated by SDS–PAGE. Proteins were transferred onto PVDF membranes, blocked, and incubated overnight at 4 °C with primary antibodies against positive exosomal markers (CD81, CD63, CD9) and the negative control marker Calnexin and Albumin (all diluted 1:1,000). After washing, membranes were incubated with HRP-conjugated secondary antibodies and visualized using the AI 600 imaging system. Band intensity was quantified using ImageJ software (NIH), and relative protein levels were analyzed to assess marker enrichment.

### 2.7 Real-time quantitative reverse transcription PCR (qRT-PCR)

Total RNA was extracted using TRIzol reagent and reverse-transcribed with a miRNA Reverse Transcription Kit (5× PrimeScript RT Mix). qPCR was performed to quantify gene expression levels, and relative expression was calculated using the 2^−ΔΔCt method. For the quantification of circRNA, GAPDH served as the endogenous reference gene. For miR-375, U6 small nuclear RNA (snRNA) was used as the endogenous control to ensure normalization accuracy. The stability of these reference genes was verified across all experimental groups. All primer sequences are listed in Supplementary Table S1.

### 2.8 Construction of Aβ1-42-induced SH-SY5Y Cell models

SH-SY5Y cells were exposed to increasing concentrations of Aβ1-42 (1, 2.5, 5, and 10 μmol/L) for 24 h to establish an *in vitro* AD-related cytotoxicity model. After treatment, cell viability and morphological alterations were evaluated using an inverted phase-contrast microscope.

### 2.9 Cell viability assay

SH-SY5Y cells were seeded into 96-well plates at a density of 3 × 10^3 cells per well and treated with Aβ1-42 for 24 h. Subsequently, 10 μL of enhanced CCK-8 reagent was added to each well, followed by a 1-h incubation at 37 °C. Absorbance was measured at 450 nm to evaluate cell viability.

### 2.10 Cell proliferation assay

Cell proliferation was evaluated using the BeyoClick™ EdU-594 Kit (Beyotime, C0078S). SH-SY5Y cells were incubated with 10 μM EdU for 2 h, fixed with 4% paraformaldehyde, and stained according to the manufacturer’s instructions. Fluorescence signals were imaged and quantified using ImageJ software.

### 2.11 Apoptosis detection

Apoptosis in SH-SY5Y cells was assessed using the Annexin V–FITC/7-AAD staining method. Cells were resuspended at a concentration of 1–5 × 10^6 cells/mL, stained with Annexin V–FITC and 7-AAD, and analyzed by flow cytometry (10,000 events per sample). Apoptotic populations were quantified using CellQuest software.

### 2.12 Plasmid and siRNA transfection

Plasmid DNA and siRNA transfections were performed using Lipofectamine™ 3,000 (Thermo Fisher Scientific) following the manufacturer’s protocol. Briefly, 5 μL of Lipofectamine™ 3,000 was diluted in 125 μL of Opti-MEM medium. Separately, 2.5 μg of plasmid DNA or 100 pmol of siRNA was diluted in 125 μL of Opti-MEM. The two solutions were mixed at a 1:1 ratio and incubated for 10–15 min at room temperature to allow complex formation. The transfection mixture was then added to cells and incubated for 4–6 h before replacing with fresh medium.

### 2.13 Biotin-RNA pull-down assay

Cells were transfected with a biotin-labeled hsa_circ_0093884 probe or a negative control probe (200 nM). After 48 h, cells were cross-linked with 1% formaldehyde for 10 min and lysed by ultrasonication at 40% amplitude for 10 min. Lysates were centrifuged at 10,000 × g for 10 min, and the supernatants were incubated with pre-washed streptavidin magnetic beads at 4 °C for 2 h with rotation. After bead washing, cross-linking was reversed by incubation at room temperature for 2 h. RNA bound to the beads was extracted using TRIzol reagent. miRNA reverse transcription was performed using the Bioindustry B532451 miRNA First-Strand cDNA Synthesis Kit, and the enrichment of miR-375 was quantified by qPCR using the Bioindustry B532461 miRNA Fluorescence PCR Kit.

### 2.14 Dual-luciferase reporter gene assay

HEK293T cells were co-transfected with the SIX4 3′UTR dual-luciferase reporter plasmid and miR-375 mimics or negative control mimics. After 24-48 h, cells were lysed on ice and centrifuged to collect the supernatant. Firefly and Renilla luciferase activities were measured using the Yisheng Dual-Luciferase Reporter Assay Kit on an LB960 chemiluminescence detector. Relative luciferase activity was calculated as the ratio of firefly to Renilla luciferase signals.

### 2.15 Statistical methods

Data distribution was assessed using the Shapiro–Wilk test. For normally distributed variables with homogenous variance, one-way ANOVA followed by Dunnett’s *post hoc* test was used for group comparisons. For non-normally distributed or heteroscedastic variables, the Kruskal–Wallis test was applied. Pearson correlation analysis was performed for normally distributed quantitative variables, while Spearman correlation was used for non-normally distributed data. In the univariate logistic regression analysis, a threshold of P < 0.10 was deliberately chosen to screen for potential predictors. According to the purposeful selection method described by Hosmer and Lemeshow (Hosmer Jr et al., 2013), a looser threshold ensures that potentially relevant confounding factors are not prematurely excluded before multivariable modeling. Subsequently, multivariable logistic regression was used to identify independent diagnostic predictors of AD. The diagnostic performance of baPWV, exosomal circRNAs, and the combined model was evaluated using receiver operating characteristic (ROC) curve analysis. To validate the stability of the model, internal validation was performed using the Bootstrap method with 1,000 resamplings. Statistical significance was set at p < 0.05. Statistical analyses were performed using SPSS Statistics 29.0. Figures were generated using GraphPad Prism 9.0.

## 3 Results

### 3.1 Baseline characteristics and intergroup comparisons

A total of 125 participants were included in the analysis, comprising 58 patients with AD, 31 with MCI, and 36 with NCI. The median age of the AD group was 91.5 years (IQR 88–95), and 46 individuals (79.3%) were male. Most participants presented with vascular risk factors, including coronary artery disease in 52 (89.7%), hypertension in 48 (82.8%), and diabetes mellitus in 27 (46.6%). No significant differences were observed among the three groups with respect to baseline demographic characteristics or comorbidities (all P > 0.05). However, diastolic blood pressure differed significantly among the three groups (P = 0.045), with a significant difference detected between the MCI and AD groups (P < 0.05).

baPWV was markedly higher in the AD group (2,545.54 ± 560.6 cm/s) compared with both the MCI and NCI groups (P < 0.001). Cognitive and geriatric assessments—including MMSE total score, attention and calculation subscore, Barthel Index, SPPB, and PHQ-9—demonstrated a progressive decline from NCI to MCI to AD (all P < 0.05). Post hoc analyses further confirmed significant intergroup differences in baPWV, orientation, registration, PHQ-9, and PSQI between the AD and MCI groups, as well as between the AD and NCI groups (all P < 0.05) (Table 1).

**TABLE 1:** Characteristics of study population classified by cognition.

Variables	NCI(n = 36)	MCI(n = 31)	AD (n = 58)	χ^2^/F	*p*
Age, y	90.5 (88,93)	91.5 (90,95.5)	91.5 (88,95)	2.988	0.224
Male/Female	33/3	24/7	46/12	3.044	0.218
BMI	22.89 ± 2.26	21.97 ± 3.38	21.97 ± 2.82	0.923	0.401
Education duration, y	12.03 ± 2.02	11.60 ± 2.90	11.08 ± 2.83	1.330	0.268
Coronary heart disease, n,%	32 (88.9)	28 (90.3)	52 (89.7)	0.037	0.982
Hypertention, n,%	31 (86.1)	27 (87.1)	48 (82.8)	0.362	0.834
Diabetes, n,%	11 (30.6)	14 (45.2)	27 (46.6)	2.555	0.279
SBP, mmHg	132.71 ± 19.39	127.21 ± 18.64	131.72 ± 19.48	0.780	0.461
DBP, mmHg	67.5 (55.5,75)	62.5 (57.5,69)	69.75 (63,74)	3.188	0.045
HR, beats/min	74.58 ± 18.37	70.42 ± 13.01	76.12 ± 12.7	1.548	0.217
Homocysteine, umol/L	16.5 ± 7.03	15.67 ± 5.29	14.8 ± 6.36	0.787	0.457
Triglyceride, mmol/L	0.65 (0.56,1.88)	1.09 (0.67,1.58)	0.89 (0.73,1.11)	0.593	0.743
HDL-C, mmol/L	1.07 ± 0.25	1.08 ± 0.29	1.01 ± 0.27	0.629	0.535
LDL-C, mmol/L	2.05 ± 0.64	2.02 ± 0.61	2.18 ± 0.74	0.763	0.468
Calcium, mmol/L	2.16 ± 0.15	2.09 ± 0.28	2.14 ± 0.15	1.126	0.328
ABI	1.14 (1.08,1.22)	1.17 (0.87,1.30)	1.11 (0.95,1.24)	0.667	0.717
baPWV, cm/s	2,149.26 ± 444.1	2,158.89 ± 459.99	2,545.54 ± 560.6	9.345	<0.001
Carotid intima-media thickness, mm	0.75 (0.73,0.9)	0.8 (0.71,0.9)	0.85 (0.76,0.98)	4.352	0.114
MMSE	28 (28,28)	25 (23,25.75)	19.5 (13.25,21)	104.554	<0.001
Orientation	10 (10,10)	10 (10,10)	8 (5.5,10)	50.617	<0.001
Registration	3 (3,3)	3 (3,3)	3 (2,3)	13.029	0.001
Attention and calculation	4 (4,5)	2 (1,3)	1 (0,1)	66.110	<0.001
Recall	2 (1,2.75)	2 (1,2)	0 (0,0)	82.468	<0.001
Language	8 (8,8)	7 (7,8)	7 (6.75,8)	13.585	0.001
Visuo constructional skills	1 (1,1)	1 (0,1)	0 (0,0)	95.532	<0.001
MoCA	27 (26,28)	20 (18,22)	14 (11.75,17)	97.692	<0.001
CDR	0 (0,0)	0.5 (0.5,0.5)	2 (1,2)	113.032	<0.001
Barthel index	100 (60,100)	77.5 (65,90)	65 (60,100)	24.941	<0.001
NRS2002	2 (1,2)	2 (2,2)	2 (2,2)	3.048	0.218
PSQI	10 (10,12)	9 (3,11)	13 (6.25,13)	12.516	0.062
FRAIL	1 (1,3)	3.5 (3,4)	3 (2,4)	38.100	<0.001
SPPB	12 (8,12)	8 (6,8)	6 (4,12)	20.936	<0.001
PHQ-9	1 (1,5)	4 (4,4)	6 (3,8.75)	9.314	0.009
GAD-7	0 (0,0)	1 (1,3)	1 (0,2)	21.290	<0.001

Abbreviations: NCI, non-cognitive impairment; MCI, mild cognitive impairment; AD, Alzheimer’s disease; BMI, body mass index; SBP, systolic blood pressure; DBP, diastolic blood pressure; HDL-C, high-density lipoprotein cholesterol; LDL-C, low-density lipoprotein cholesterol; ABI, ankle-brachial index; baPWV, brachial-ankle pulse wave velocity; MoCA, montreal cognitive assessment; MMSE, Mini-Mental State Examination; CDR, clinical dementia rating; ADL, activities of daily living; SPPB, short physical performance battery; MNA, mini nutritional assessment; PSQI, pittsburgh sleep quality index; PHQ-9, Patient Health Questionnaire-9; GAD-7, Generalized Anxiety Disorder-7.

### 3.2 Correlation analysis between baPWV and clinical characteristics

baPWV was positively correlated with age, systolic and diastolic blood pressure, serum calcium, Clinical Dementia Rating (CDR) score, PSQI score, and PHQ-9 score (P < 0.05). In contrast, baPWV showed negative correlations with Moca score, MMSE total score, orientation subscale, recall subscale, visuoconstructional subscale, and SPPB score (P < 0.05) (Table 2).

**TABLE 2:** Correlation analysis of baPWV and clinical features.

Variables	Age	SBP	DBP	Carotid intima-media thickness	Homocysteine	Triglyceride	Calcium	MoCa
r	0.198	0.234	0.325	−0.003	0.004	0.161	0.216	−0.307
p	0.028	0.009	<0.001	0.977	0.969	0.073	0.016	<0.001

Variables	CDR	MMSE	Orientation	Registration	Attention and calculation	Recall	Language	Visuo constructional skills
r	0.354	−0.316	−0.238	−0.032	−0.173	−0.296	−0.044	−0.208
*p*	<0.001	<0.001	0.008	0.725	0.054	0.001	0.624	0.020

Variables	Barthel index	NRS 2002	PSQI	FRAIL	SPPB	PHQ-9	GAD-7
r	−0.137	−0.097	0.222	0.112	−0.405	0.261	−0.028
*p*	0.127	0.310	0.017	0.229	<0.001	0.004	0.767

Abbreviations: baPWV, brachial-ankle pulse wave velocity; SBP, systolic blood pressure; DBP, diastolic blood pressure; MoCA, montreal cognitive assessment; CDR, clinical dementia rating; MMSE, Mini-Mental State Examination; NRS, 2002, Nutritional Risk Screening 2002; PSQI, pittsburgh sleep quality index; FRAIL, fatigue, Resistance, Ambulation, Illnesses, and Loss of Weight; SPPB, short physical performance battery; PHQ-9, Patient Health Questionnaire-9; GAD-7, Generalized Anxiety Disorder-7.

### 3.3 Characterization of EPC-Exos

**FIGURE 1:**
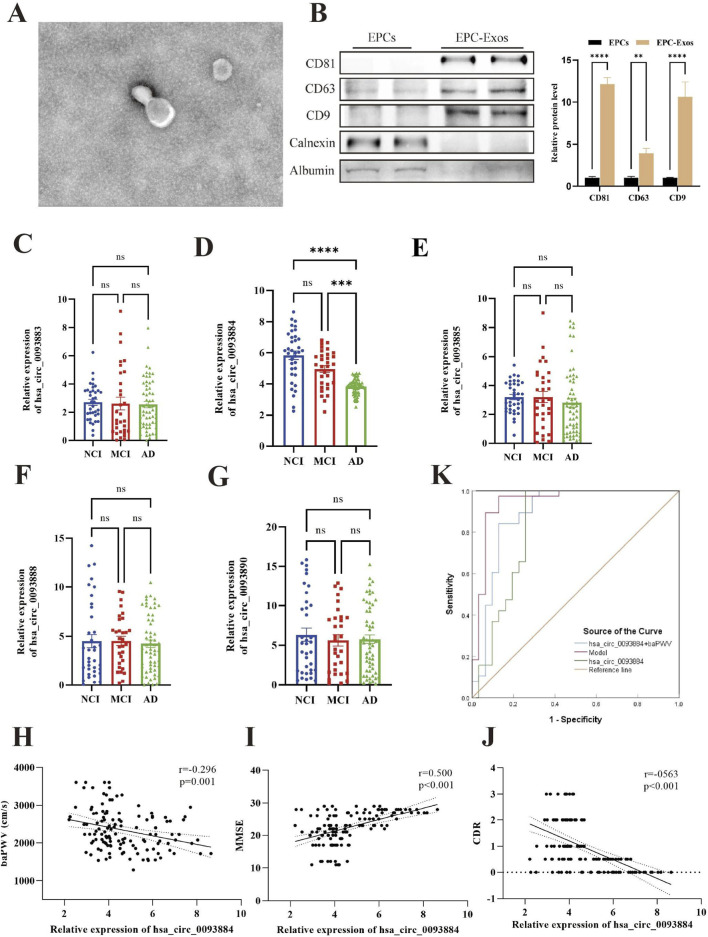
Characterization of CD34^+^ progenitor-derived exosomes (EPC-Exos) and clinical validation. **(A)** Morphology of exosomes under transmission electron microscopy. **(B)** Western blot validation of exosomal purity and enrichment. Left panel: Representative blots showing the expression of positive markers (CD81, CD63, CD9) and the negative control (Calnexin and Albumin) in cell lysates (CL) versus EPC-Exos. Right panel: Semi-quantitative densitometric analysis of marker protein levels (*P* < 0.05). **(C–G)** Comparison of the expression levels of SIRT1-derived circRNAs in plasma EPC-Exos among the groups. **(H–J)** Correlation of plasma EPC-Exos hsa_circ_0093884 expression levels with baPWV, MMSE, and CDR scores. **(K)** ROC curve analysis evaluating the diagnostic value of plasma EPC-Exos hsa_circ_0093884 and the combined model for Alzheimer’s disease.

Transmission electron microscopy showed that CD34^+^ progenitor-derived exosomes (EPC-Exos) exhibited the characteristic cup-shaped morphology with well-defined bilayer membranes (Figure 1A). Nanoparticle tracking analysis indicated a mean particle diameter of 107.9 ± 12.3 nm, consistent with the expected size range of exosomes. Western blot analysis confirmed the enriched expression of the exosomal markers CD81, CD63, and CD9 in the isolated fractions. In contrast, the endoplasmic reticulum marker Calnexin and the highly abundant plasma protein Albumin were detectable in cell lysates/plasma but absent in EPC-Exos, confirming the high purity of the isolation and the successful depletion of common protein contaminants. Semi-quantitative densitometric analysis further verified significantly higher levels of CD81, CD63, and CD9 in EPC-Exos compared to cell lysates (P < 0.05), demonstrating the efficiency of the enrichment method (Figure 1B).

### 3.4 Comparison of SIRT1-Derived circRNA levels in plasma EPC-Exos

Among the five SIRT1-derived circRNAs quantified in plasma EPC-Exos (hsa_circ_0093883, hsa_circ_0093884, hsa_circ_0093885, hsa_circ_0093888, hsa_circ_0093890), hsa_circ_0093884 showed a significant reduction in the AD group (3.88, 3.44–4.12) compared with both MCI (5.22, 3.97–5.89) and NCI (6.07, 4.70–7.02) (P < 0.001) (Figures 1C–G). Post hoc Dunn’s tests further demonstrated a stepwise decrease from NCI to MCI to AD (AD vs. NCI: P < 0.001; AD vs. MCI: P = 0.003).

### 3.5 Correlation between plasma EPC-Exos hsa_circ_0093884 and clinical characteristics

Spearman correlation analysis revealed that plasma hsa_circ_0093884 levels were positively associated with MMSE total score, orientation, attention and calculation, recall, visuoconstructional subscales, Barthel Index, and SPPB scores (all P < 0.05). Conversely, hsa_circ_0093884 levels were negatively associated with baPWV, CDR score, PSQI score, FRAIL score, and PHQ-9 score (all P < 0.05) (Figures 1H–J; Table 3).

**TABLE 3:** Correlation analysis of hsa_circ_ 0093884 and clinical features.

Variables	Age	SBP	DBP	Carotid intima-media thickness	Homocysteine	Triglyceride	Calcium
r	−0.08	0.026	−0.009	0.16	0.148	0.116	0.001
p	0.377	0.777	0.918	0.15	0.106	0.2	0.993

Variables	Orientation	Registration	Attention and calculation	Recall	Language	Visuo constructional skills	Barthel index
r	0.367	0.137	0.343	0.402	0.12	0.5	0.27
*p*	<0.001	0.128	<0.001	<0.001	0.181	<0.001	0.002

Variables	NRS 2002	PSQI	FRAIL	SPPB	PHQ-9	GAD-7
r	−0.049	−0.227	−0.219	0.256	−0.18	−0.07
*p*	0.604	0.015	0.018	0.007	0.047	0.458

Abbreviations: SBP, systolic blood pressure; DBP, diastolic blood pressure; NRS, 2002, Nutritional Risk Screening 2002; PSQI, pittsburgh sleep quality index; FRAIL, fatigue, Resistance, Ambulation, Illnesses, and Loss of Weight; SPPB, short physical performance battery; PHQ-9, Patient Health Questionnaire-9; GAD-7, Generalized Anxiety Disorder-7.

### 3.6 Risk factor analysis for Alzheimer’s disease

Univariate logistic regression identified baPWV, plasma EPC-Exos hsa_circ_0093884 levels, multi-vascular risk burden (≥2 comorbidities: coronary artery disease, hypertension, diabetes), and CGA scores as potential factors associated with AD (P < 0.10). Variables with P < 0.10 were entered into a multivariable logistic regression model, which revealed that baPWV and plasma hsa_circ_0093884 were independent factors associated with AD (P < 0.05) (Table 4).

**TABLE 4:** Univariate and multivariate logistic regression analyses of factors associated with AD.

Variables	Univariate analysis			Multivariate analysis		
	OR	95%CI	*p*	OR	95%CI	*p*
hsa_circ_ 0093884	0.265	0.161,0.436	<0.001	0.261	0.109,0.626	0.003
baPWV	1.002	1.001,1.002	<0.001	1.002	1.000,1.004	0.045
Age	1.019	0.950,1.093	0.592	​	​	​
Gender	1.487	0.590,3.749	0.400	​	​	​
History of two or more vascular- related risks	0.955	0.359,2.540	0.927	​	​	​
Homocysteine	0.967	0.912,1.025	0.255	​	​	​
Triglyceride	0.970	0.506,1.861	0.928	​	​	​
Calcium	0.757	0.204,8.912	0.757	​	​	​
Carotid intima-media thickness	0.062	0.002,1.629	0.095	0.006	0.000,3.011	0.106
Barthel index	0.962	0.943,0.981	<0.001	0.940	0.853,1.036	0.211
FRAIL	1.313	0.924,1.865	0.128	​	​	​
SPPB	0.825	0.722,0.943	0.050	1.257	0.645,2.451	0.502
PSQI	1.138	1.020,1.259	0.012	1.049	0.793,1.388	0.737
GAD-7	1.037	0.736,1.460	0.837	​	​	​
PHQ-9	1.155	1.022,1.306	0.021	1.551	1.215,1.980	0.082

Abbreviations: AD, Alzheimer’s disease; OR, odds ratio; CI, confidence interval; baPWV, brachial-ankle pulse wave velocity; ADL, activities of daily living; SPPB, short physical performance battery; MNA, mini nutritional assessment.

### 3.7 Diagnostic value of plasma EPC-Exos hsa_circ_0093884 and baPWV for Alzheimer’s disease

Binary logistic regression analysis was used to construct ROC curves for plasma EPC-Exos hsa_circ_0093884 levels, baPWV, and the predictive probability derived from the multivariate logistic regression model. The area under the curve (AUC) for plasma EPC-Exos hsa_circ_0093884 was 0.818 (95% CI 0.738-0.898, P < 0.001). Combining baPWV with plasma EPC-Exos hsa_circ_0093884 increased the AUC to 0.863 (95% CI, 0.794–0.933; P < 0.001). The multivariate logistic regression model achieved an AUC of 0.943 (95% CI, 0.881–1.000; P < 0.001), with a maximum Youden index of 0.845, corresponding to a sensitivity of 97.4% and specificity of 87.1% (Figure 1K). To validate the stability of the model, internal validation was performed using the Bootstrap method with 1,000 resamplings. The analysis confirmed that the key predictors, specifically hsa_circ_0093884 (Bootstrap *P* = 0.003, 95% CI: 3.063–1.104) and baPWV (Bootstrap *P* = 0.024), maintained statistical significance, supporting the robustness of the diagnostic model. Collinearity diagnostics revealed that the Variance Inflation Factor (VIF) for all included predictors ranged from 1.083 to 1.978, indicating no significant multicollinearity.

### 3.8 Aβ1-42–induced AD-like injury in SH-SY5Y cells

**FIGURE 2:**
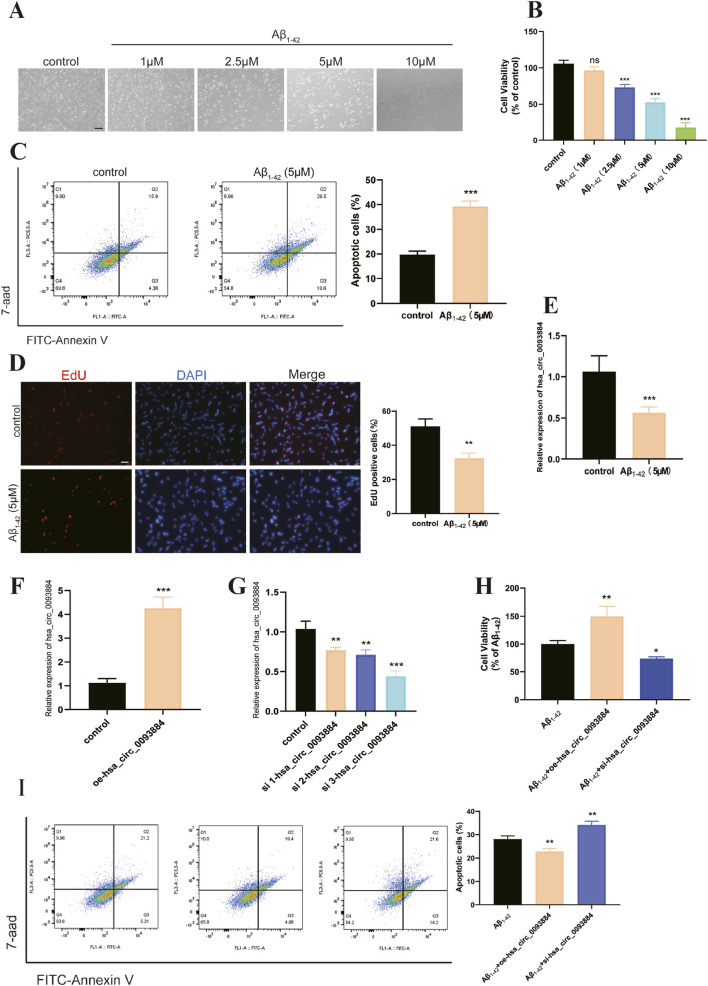
**(A)** Morphological changes in SH-SY5Y cells observed under a microscope after 24-h treatment with different concentrations of Aβ1-42 (1 μmol/L, 2.5 μmol/L, 5 μmol/L, 10 μmol/L). Scale bar = 100 μM. **(B)** CCK8 assay to determine the effect of different concentrations of Aβ1-42 on the viability of SH-SY5Y cells. **(C)** Annexin V-FITC/7-AAD dual staining to compare the apoptosis between the control group and the 5 μmol/L Aβ1-42-induced group. **(D)** EdU cell proliferation assay to compare the proliferation between the control group and the 5 μmol/L Aβ1-42-induced group. Scale bar = 50 Μm. **(E)** Changes in the expression of hsa_circ_0093884 in cells after Aβ1-42 modeling. **(F)** Overexpression efficiency of the hsa_circ_0093884 overexpression plasmid in SH-SY5Y cells. **(G)** Inhibition efficiency of the hsa_circ_0093884 siRNA in SH-SY5Y cells. **(H)** CCK8 assay to detect the effects of hsa_circ_0093884 overexpression/inhibition on cell viability. **(I)** Annexin V-FITC/7-AAD dual staining to compare the effects of hsa_circ_0093884 overexpression/inhibition on cell apoptosis (*P < 0.05, **P < 0.01, ***P < 0.001).

Exposure to Aβ1-42 (1–10 μM) induced a dose-dependent reduction in SH-SY5Y cell viability. Treatment with 5 μM Aβ1-42 decreased cell viability by 48.2% relative to controls (P < 0.001; Figure 2B) and was accompanied by marked morphological deterioration, including cell shrinkage and neurite retraction (Figure 2A). Flow cytometry revealed a two-fold increase in apoptotic cells (39.30% ± 2.14% vs. 19.76% ± 1.43% in controls; P < 0.001; Figure 2C). Consistently, EdU incorporation assays demonstrated a 36.5% reduction in proliferating cells (32.43% ± 2.94% vs. 51.01% ± 4.41%; P = 0.002; Figure 2D).

### 3.9 Dysregulation and functional role of hsa_circ_0093884 in AD Cell models

hsa_circ_0093884 expression was markedly reduced in Aβ1-42-treated SH-SY5Y cells compared with controls (P < 0.001; Figure 2E). Efficient modulation of hsa_circ_0093884 was achieved through plasmid overexpression and siRNA-mediated knockdown, with siRNA3 exhibiting the strongest suppressive effect (Figures 2F,G). Functionally, overexpression of hsa_circ_0093884 significantly enhanced cell viability (P < 0.001) and attenuated apoptosis (P = 0.002; Figures 2H,I). In contrast, siRNA3-mediated knockdown aggravated Aβ1-42-induced neuronal injury, further reducing viability (P < 0.001) and increasing apoptosis (P = 0.004; Figures 2H,I). These findings suggest that hsa_circ_0093884 exerts a protective role against Aβ1-42–induced neurotoxicity.

### 3.10 hsa_circ_0093884 regulates the miR-375/SIX4 axis in AD Cell models

**FIGURE 3:**
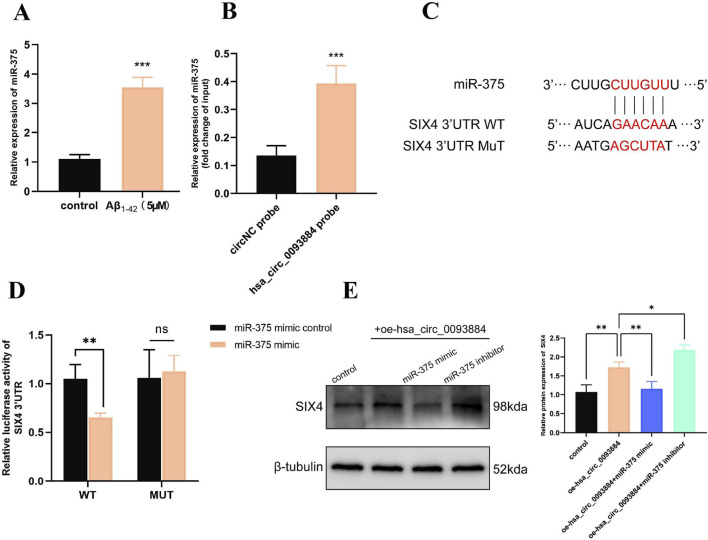
**(A)** Detection of miR-375 expression level in Aβ1-42-modeled cells. **(B)** miR-375 was highly enriched in the RNA pulled down by the biotinylated hsa_circ_0093884 probe **(A)**. **(C)** Bioinformatics analysis indicates that the 3′UTR of SIX4 contains a binding site for miR-375. **(D)** Dual-luciferase reporter assay verifies the targeting binding of miR-375 and the 3′UTR of SIX4. **(E)** Western blot analysis of the changes in SIX4 protein expression after co-transfection of hsa_circ_0093884 and miR-375 mimic/inhibitor. (*P < 0.05, **P < 0.01, ***P < 0.001).

Bioinformatics analyses identified several candidate miRNAs downstream of hsa_circ_0093884, among which miR-375 showed the strongest relevance to AD. Aβ1-42–treated SH-SY5Y cells displayed significant upregulation of miR-375 (P < 0.001; Figure 3A). Biotinylated RNA pull-down assays confirmed direct interaction between hsa_circ_0093884 and miR-375, with the circRNA probe markedly enriching miR-375 relative to controls (P < 0.001; Figure 3B), indicating that hsa_circ_0093884 functions as a miR-375 sponge. Further analysis revealed a predicted miR-375 binding site within the SIX4 3′UTR (Figure 3C), which was validated by dual-luciferase reporter assays using wild-type and mutant constructs (Figure 3D). Functionally, hsa_circ_0093884 overexpression increased SIX4 protein levels, an effect partially reversed by miR-375 mimics, whereas miR-375 knockdown further enhanced SIX4 expression (Figure 3E). These results demonstrate that hsa_circ_0093884 modulates the AD cell phenotype through the miR-375/SIX4 regulatory pathway.

## 4 Discussion

This study identifies elevated baPWV and reduced levels of circulating CD34^+^ progenitor-derived exosomal hsa_circ_0093884 as independent factors associated with AD. These findings align with the growing recognition that vascular aging contributes to AD pathogenesis, potentially by impairing cerebral perfusion and disrupting the neurovascular unit (Custodia et al., 2021; Maler et al., 2006). Consistent with our results, vascular lesions are observed in a significant proportion of AD brains, and endothelial deterioration often precedes overt macrovascular pathology (Esiri et al., 2014). Transcriptomic analyses also highlight endothelial pathways as dysregulated in AD brain tissue (Lau et al., 2020), while animal studies indicate that vascular dysfunction may occur prior to amyloid deposition (Meyer et al., 2008). Our data extend these observations by suggesting that peripheral vascular markers (baPWV and EPC-Exos) may reflect these central pathological changes.

The inverse correlation observed between baPWV and cognitive performance underscores the potential link between systemic arterial stiffness and neurodegeneration. Prior studies demonstrate that baPWV is independently associated with Aβ burden (Hughes et al., 2013), with each standard-deviation increase doubling the odds of Aβ positivity in cognitively normal older adults and quadrupling the risk over 2 years (Hughes et al., 2014). Elevated baPWV also correlates with reduced volumes of AD-vulnerable regions such as the hippocampus and entorhinal cortex (Jochemsen et al., 2015). Advanced imaging techniques, including 4D-flow MRI, have revealed similarly heightened intracranial PWV in AD patients, with younger AD subjects displaying vascular aging patterns resembling those of significantly older individuals (Rivera-Rivera et al., 2021). Collectively, these findings support the hypothesis that systemic vascular dysfunction may serve as a mechanistic bridge linking cardiovascular aging to clinical progression in AD, although the precise temporal relationship remains to be fully elucidated in longitudinal settings.

At the molecular level, exosomes have emerged as potential messengers in the crosstalk between the peripheral circulation and the central nervous system. We identified CD34^+^ progenitor-derived exosomal hsa_circ_0093884 as a dysregulated molecule in AD plasma. Its progressive downregulation in the plasma of AD patients and in Aβ1–42-treated SH-SY5Y cells mirrors endothelial impairment-a hallmark of vascular aging (Romaus-Sanjurjo et al., 2023). These extracellular vesicles are capable of crossing the blood-brain barrier, supporting the existence of a “vascular-neuronal axis” wherein peripheral vascular factors may modulate neuronal viability (Akhter, 2018). In our *in vitro* experiments, hsa_circ_0093884 functioned as a sponge for miR-375, thereby relieving the suppression of the transcription factor SIX4. Overexpression of hsa_circ_0093884 restored SIX4 expression and enhanced neuronal survival, whereas knockdown exacerbated Aβ-induced cytotoxicity. These results are consistent with clinical observations implicating elevated miR-375 levels in AD cerebrospinal fluid and their correlation with Aβ burden (Denk et al., 2015), and with evidence that SIX4 promotes cell survival through regulation of Bcl-X (Konishi et al., 2006). This competitive endogenous RNA axis provides a mechanistic explanation for the neuroprotective effects associated with hsa_circ_0093884. This focused *in vitro* approach was essential to isolate the specific biological role of hsa_circ_0093884 from the numerous systemic confounding factors present in AD patients. By demonstrating that the direct modulation of this circRNA can dictate neuronal survival under Aβ toxicity, we establish that hsa_circ_0093884 is not merely a passive byproduct of vascular aging, but a functional modulator with a clear mechanistic link to the neurodegenerative process.”

Together, these findings support a comprehensive model that integrates vascular assessment (baPWV), circulating circRNA quantification (hsa_circ_0093884), and geriatric functional evaluation. This multidimensional approach captures vascular pathology, molecular dysregulation, and clinical manifestations simultaneously, offering a clinically actionable framework for early detection and risk stratification. The observed associations between vascular biomarkers and geriatric domains further highlight the multisystem impact of vascular aging. The inverse relationship between hsa_circ_0093884 and PHQ-9 scores echoes evidence that endothelial dysfunction disrupts mood-related frontal-subcortical circuits (Waclawovsky et al., 2021). Elevated baPWV among individuals with poor sleep quality is consistent with findings linking sleep fragmentation to microvascular damage (Saz-Lara et al., 2022). Moreover, associations between SPPB performance and vascular biomarkers suggest shared biological pathways between sarcopenia and endothelial decline (Gonzales et al., 2015; Proctor and Parker, 2006). These correlations reinforce the concept of vascular aging as a unifying mechanism connecting cognitive decline with common geriatric syndromes, suggesting that a multidimensional evaluation approach is valuable for the clinical management of older adults with cognitive impairment.

Several limitations warrant consideration. First, the cross-sectional design precludes causal inference. While we observed strong associations, we cannot determine whether vascular dysfunction and circRNA dysregulation are upstream drivers or downstream consequences of AD pathology. Second, although we enriched for CD34^+^ exosomes to target the endothelial progenitor lineage, CD34 is also expressed on hematopoietic stem cells (HSCs). Therefore, the exosomes analyzed likely represent a mixed population of progenitor-derived vesicles rather than a purely endothelial-specific fraction. Third, regarding the mechanistic pathway, while we validated the hsa_circ_0093884/miR-375/SIX4 axis, the downstream targets of SIX4 were not experimentally verified in this study but were inferred from existing literature (Konishi et al., 2006). Furthermore, while our *in vitro* model confirmed the downstream intracellular signaling of the proposed axis, the dynamic transport kinetics across the BBB and the systemic interactions in a multi-organ environment require further investigation. These cellular experiments represent a successful preliminary validation of the functional ‘proof-of-concept.’ Future research utilizing AD transgenic mouse models will be instrumental in mapping the full *in vivo* trajectory of these vascular-derived exosomes and exploring their potential as therapeutic targets. Finally, the sample size was relatively limited, and the study population consisted of geriatric inpatients with multiple comorbidities. This may limit the generalizability of our findings to community-dwelling populations or younger AD cohorts. Future longitudinal studies with larger sample sizes and *in vivo* models are needed to validate these findings and explore the therapeutic potential of this vascular-neuronal axis.

## 5 Conclusion

In conclusion, this study identifies elevated baPWV and reduced levels of CD34^+^ progenitor-derived exosomal hsa_circ_0093884 as valuable diagnostic biomarkers associated with Alzheimer’s disease. Our findings support a potential vascular-neuronal axis, partially mediated by the hsa_circ_0093884/miR-375/SIX4 regulatory pathway, which may link vascular aging to neurodegeneration. Furthermore, integrating these vascular markers with Comprehensive Geriatric Assessment offers a multidimensional approach for the clinical evaluation of cognitive impairment. Future longitudinal studies are warranted to validate causal relationships and explore the therapeutic potential of targeting this axis in AD.

## Data Availability

The datasets presented in this study can be found in online repositories. The names of the repository/repositories and accession number(s) can be found in the article/Supplementary Material.
